# Association Between Hormonal Contraceptive Use and Lipedema: A Cross-Sectional Study With 637 Brazilian Women

**DOI:** 10.7759/cureus.99189

**Published:** 2025-12-14

**Authors:** Alexandre C Amato, Juliana L Amato, Daniel Benitti

**Affiliations:** 1 Vascular Surgery, Amato - Instituto de Medicina Avançada, São Paulo, BRA; 2 Gynecology, Amato - Instituto de Medicina Avançada, São Paulo, BRA; 3 Vascular and Endovascular Surgery, Medical Valens Center, São Paulo, BRA

**Keywords:** cross-sectional study, hormonal contraceptives, hormones, lipedema, quality of life

## Abstract

Background: Lipedema is a chronic, progressive adipose tissue disorder that predominantly affects women and is characterized by disproportionate fat accumulation, pain, and edema. Hormonal fluctuations are frequently reported as triggers or modulators of symptoms, but the impact of exogenous hormones, especially hormonal contraceptives, remains poorly defined.

Objective: This study aimed to investigate the association between hormonal contraceptive use and the presence, severity, and self-reported worsening of lipedema symptoms in Brazilian women.

Methods: This observational, cross-sectional study was conducted at Amato - Instituto de Medicina Avançada using a structured online questionnaire applied between August and November 2025. We included women aged 18 years or older, residing in Brazil, with suspected or confirmed lipedema who provided electronic consent and completed core sections on lipedema symptoms, hormonal history, and contraceptive use. Questionnaires with less than 50% of core items answered, duplicate entries, and biologically implausible values were excluded. Symptom (0-8) and quality of life (0-15) scores were calculated. Self-reported changes in symptoms after starting hormonal contraceptives were analyzed as a four-level variable and as a binary worsening variable. Free text on side effects and timing of onset was categorized with natural language processing. Statistical analyses included chi-squared tests, Spearman correlations, and logistic and linear regression.

Results: A total of 637 women were included (mean age 41.8±8.7 years; mean body mass index (BMI) 28.9±6.4 kg/m²); 77.1% had a confirmed diagnosis of lipedema and 92.3% were current or previous users of hormonal contraceptives. Among users, 58.8% reported symptom worsening after starting contraceptives (34.5% severe; 24.3% slight), 40.3% reported no change, and 0.9% reported improvement (p<0.001). Free text analysis showed that 15.1% reported onset of lipedema symptoms temporally coinciding with contraceptive initiation. In multivariable analysis, a higher baseline symptom score was the strongest independent predictor of worsening, while duration of contraceptive use was not associated with risk. Pain intensity and BMI were the main independent predictors of quality of life impact.

Conclusions: In this large sample of Brazilian women with suspected or confirmed lipedema, hormonal contraceptive use was frequently associated with self-reported worsening of symptoms, and a substantial minority reported symptom onset around contraceptive initiation. Women with higher baseline symptom burden appeared particularly vulnerable. These findings support individualized contraceptive counseling for women with lipedema and highlight the need for prospective studies with objective measures to clarify causality and mechanisms.

## Introduction

Lipedema is a chronic, progressive disorder of the subcutaneous adipose tissue that occurs almost exclusively in women and is characterized by symmetrical, painful fat accumulation in the limbs, accompanied by edema, increased sensitivity, and resistance to weight loss [1,2]. With an estimated prevalence of 12.3% among women, lipedema imposes a substantial burden on quality of life and functional capacity in those affected [3].

The etiology of lipedema remains incompletely elucidated. Evidence suggests a genetic component and a modulating role of female sex hormones, as the disease frequently manifests or worsens during periods of hormonal fluctuation (puberty, pregnancy, menopause). Particularly, hormonal contraceptives have been anecdotally associated with symptom worsening, although robust scientific evidence is scarce [4,5].

The biological plausibility of this association lies in multiple mechanisms. The estrogenic components of hormonal contraceptives may amplify mast cell activation, alter vascular permeability, compromise lymphatic function, and influence adipocyte proliferation. Additionally, common side effects such as weight gain and fluid retention may exacerbate clinical manifestations [6,7].

In Brazil, where hormonal contraceptive use is prevalent and lipedema remains underdiagnosed, understanding this relationship is of extreme clinical and public health relevance [8]. This cross-sectional study aimed to investigate the association between hormonal contraceptive use and the presence, severity, and progression of lipedema in a sample of 637 Brazilian women, utilizing an innovative methodological approach with natural language processing via large language models for qualitative data enrichment. We sought to quantify this association, identify predictors of worsening, explore intermediate mechanisms, and assess the impact on quality of life, providing evidence for safer contraceptive counseling in this vulnerable population.

## Materials and methods

This observational cross-sectional study was based on a self-administered online questionnaire and was conducted at Amato - Instituto de Medicina Avançada between August and November 2025. The target population comprised women aged 18 years or older residing in Brazil with suspected or confirmed lipedema who consented to participate electronically. Eligible participants were women who reported either a previous medical diagnosis of lipedema or compatible symptoms according to a screening questionnaire and who completed at least the core sections of the survey on lipedema symptoms, hormonal and reproductive history, and hormonal contraceptive use. Incomplete questionnaires with less than 50% of core questions answered, duplicate entries identified by IP address and time stamp, and records with clearly invalid or internally inconsistent values were excluded from the analyses, for example, impossible ages, heights, or weights for adults or combinations such as age at menarche greater than current age or negative durations of contraceptive use. Participant recruitment was carried out through the dissemination of the survey link on social media platforms, including Instagram, Facebook, and WhatsApp, through support groups for women with lipedema, specialized clinics and offices, and patient associations. After applying the inclusion and exclusion criteria, the final analytical sample consisted of 637 participants.

A structured questionnaire specifically developed for this study was used and comprised eight sections. Section A collected demographic data, including age, weight, height, and geographic location, and presented the informed consent form. Section B comprised nine lipedema screening questions that investigated leg pain, progressive swelling during the day, worsening of symptoms with heat, sensitivity to touch, easy bruising, disproportionate fat distribution, resistance of leg size to diet and exercise, family history suggestive of lipedema, and the timing of symptom onset. Section C assessed the diagnosis and clinical characteristics of lipedema and included items on medical confirmation of diagnosis, date of diagnosis, specialty of the responsible physician, lipedema stage from 1 to 4, lipedema type from I to IV, and associated comorbidities. Section D collected information on hormonal and reproductive history, including age at menarche, menstrual cycle regularity, number of pregnancies and miscarriages, menopausal status, use of hormone replacement therapy, and self-reported thyroid alterations.

Section E, which was the main focus of this study, consisted of 14 questions regarding contraceptive use. Hormonal contraceptive exposure was classified in three mutually exclusive categories: current user, defined as using any hormonal contraceptive at the time of the survey; previous user, defined as having used hormonal contraceptives in the past but having completely discontinued them; and never user. These items assessed current use status, type of contraceptive method such as pill, intrauterine device (IUD), implant, vaginal ring, or injection, commercial name of the product, current and total duration of use, age at onset of use, pattern of use classified as continuous versus interrupted, reasons for discontinuation, perceived side effects, perception of lipedema worsening after starting contraceptives, specific changes in symptoms, associated weight gain, and alternative contraceptive methods used. Reasons for discontinuation were asked only to previous users. Section F assessed the impact of lipedema on quality of life through questions on mobility limitations, difficulties in daily activities, impact on work, social and emotional impact, a visual analog scale for pain ranging from 0 to 10, sleep quality, self-esteem rated on a four-point Likert scale with the options excellent, good, fair, and low, and perception of family support. Section G investigated current treatments such as lymphatic drainage, compression stockings, physical therapy, nutritional and psychological follow-up, and lipedema surgery, as well as satisfaction with these treatments. Finally, Section H allowed for additional observations in a free-text field and asked whether participants were interested in receiving the study results. The full structure of the questionnaire, translated into English, is presented in the Appendices.

The primary outcome variable was self-reported lipedema worsening associated with hormonal contraceptive use. This was recorded on a four-category scale with the response options no difference noticed, yes slightly, yes severe worsening, and improved. For the main analyses, we also derived a binary outcome that contrasted any degree of worsening, combining the categories yes slightly and yes severe worsening, against no worsening or improvement, combining no difference noticed and improved. Secondary outcomes included a lipedema symptom score, a quality of life impact score, the pain scale, and self-esteem. The symptom score ranged from 0 to 8 and was calculated as the sum of affirmative responses to the eight screening questions in Section B that assessed pain, swelling, worsening with heat, sensitivity to touch, easy bruising, disproportionate fat accumulation, resistance of leg size to diet and exercise, and family history. Higher values indicated a greater burden of symptoms. The quality of life impact score ranged from 0 to 15 and was computed as a weighted sum of five items in Section F that evaluated mobility limitations, difficulties in daily activities, impact on work, social impact, and emotional impact, with higher scores reflecting greater functional impairment. Pain intensity was assessed on a numerical rating scale from 0, meaning no pain, to 10, meaning maximum pain. Self-esteem was assessed with a single Likert-type item as described above.

The main explanatory variables related to hormonal contraceptives included current versus previous versus never use, the type of contraceptive used, the duration of use expressed in years, and the age at onset of use. Covariates included age in years, body mass index (BMI) in kilograms per square meter, age at menarche, time since menarche, number of self-reported comorbidities, lipedema stage and type, and current treatments for lipedema.

Raw data underwent multistep processing. Initially, cleaning and validation were performed, including duplicate removal based on IP address and time stamp and consistency checks across variables. Questionnaires with extensive missingness in the core sections, defined as less than 50% of core questions answered, had already been excluded at the eligibility stage as described above. For the remaining records, missing values were handled in a conservative way. Derived variables such as the lipedema symptom score, the corresponding symptom severity categories (mild, moderate, severe, very severe), and the quality of life impact score were calculated only when all required items for each score were available and were set to missing otherwise. No data imputation was performed, and all descriptive analyses and regression models used an available case approach, so that each analysis included all participants with valid data for the variables involved.

After these steps, derived variables were calculated for use in the analyses. BMI was computed as weight divided by height squared and categorized according to World Health Organization criteria [9]. Age was grouped into five-year categories for descriptive purposes. The lipedema symptom score and the quality of life impact score were calculated as described previously. Duration of hormonal contraceptive use was originally collected as a free-text field in Section E. During data processing, these responses were parsed and converted into a numeric variable that expressed total duration of use in years, which was then used as a continuous predictor in descriptive and multivariable analyses.

To enrich the qualitative information, data enrichment was performed using natural language processing with a local language model implemented in LM Studio (Element Labs, Inc., Austin, Texas, United States), using the openai/gpt-oss-20b model. Free-text fields were processed for the automatic categorization of side effects into eight predefined classes. Weight gain referred to any self-reported increase in body weight attributed to contraceptive use. Mood swings encompassed affective symptoms such as irritability, emotional lability, anxiety, or depressed mood. Headache included reports of headache or migraine temporally associated with contraceptives. Nausea covered nausea, vomiting, or other upper gastrointestinal discomfort. Reduced libido referred to decreased sexual desire or sexual dysfunction. Swelling captured reports of edema or increased swelling of the legs or body. Bleeding referred to abnormal uterine bleeding, including spotting, breakthrough bleeding, or changes in menstrual flow pattern. Side effects that did not clearly fit into these categories, such as acne or breast tenderness, were grouped under a residual "other" category. The same approach was used to extract and classify treatments into categories such as diet, exercise, compression stockings, lymphatic drainage, medications, surgery, and physical therapy. The processing also included the identification and standardization of associated comorbidities and the temporal classification of symptom onset into categories such as puberty, pregnancy, menopause, and contraceptive use. A random sample of approximately 10% of the automatically categorized responses was manually reviewed by a clinician to ensure consistency and accuracy, and the prompts were refined iteratively when needed.

Statistical analyses were performed using Python Version 3.11 (Python Software Foundation, Wilmington, Delaware, United States) with the pandas, numpy, scipy, statsmodels, and scikit learn packages. For descriptive analyses, continuous variables were summarized as mean and standard deviation and as median and interquartile range, while categorical variables were presented as absolute and relative frequencies. The normality of distributions was assessed using the Shapiro-Wilk test. In inferential analyses, chi-squared tests were used for associations between categorical variables, Student t-tests or Mann-Whitney U tests were used for comparisons between two groups, and analysis of variance or Kruskal-Wallis tests were applied to compare three or more groups, as appropriate. Spearman correlation coefficients were calculated to assess associations between continuous variables. For multivariable analysis, a logistic regression model was fitted to identify factors associated with any worsening due to contraceptives. The dependent variable in this model was the binary worsening outcome, and independent variables included age, BMI, age at menarche, duration of contraceptive use, symptom score, and number of comorbidities. Results were presented as odds ratios with 95% confidence intervals. Additionally, a multiple linear regression model was fitted to identify factors associated with the quality of life impact score. In this model, the dependent variable was the quality of life impact score, and independent variables included age, BMI, symptom score, pain scale, degree of worsening with contraceptives, and number of comorbidities. Results were presented as standardized beta coefficients. A significance level of 5% with two-tailed tests was adopted for all analyses, and p-values were adjusted for multiple comparisons using the Bonferroni method when appropriate. All analyses were performed using an available case approach, so that each model included all participants with valid data for the relevant variables.

The study was conducted in accordance with the ethical principles of the Declaration of Helsinki and Brazilian regulations, including National Health Council resolutions 466/2012 and 510/2016. All participants provided electronic informed consent after reading the informed consent form presented at the beginning of the questionnaire. Data were anonymized and stored securely using AES 256 encryption. The study protocol was approved by the Institutional Ethics Committee of Amato - Instituto de Medicina Avançada (approval number: 90936425.0.0000.0081). The anonymized dataset and the code used to reproduce the analyses are available from the authors upon reasonable request, in compliance with Brazilian data protection guidelines.

## Results

A total of 637 women participated in this study, representing the largest Brazilian sample investigated on lipedema to date. The demographic and clinical characteristics revealed a mean age of 41.8±8.7 years (range: 19-68 years) and a mean BMI of 28.9±6.4 kg/m², with the majority of participants (77.1%; n=491) having a confirmed diagnosis of lipedema by a healthcare professional. The geographic distribution showed predominance of participants from the Southeast region (64.8%), followed by the South (16.6%), which reflects both the regional concentration of specialized lipedema care centers and the demographic distribution of the Brazilian population. Detailed demographic and clinical characteristics of the sample are presented in Table 1.

**Table 1:** Demographic and clinical characteristics of the study participants (n=637) Data are presented as mean±SD, median (IQR), and range (min-max) for continuous variables and as n (%) for categorical variables. SD: standard deviation; IQR: interquartile range; BMI: body mass index

Variable	Value
Age (years)	
Mean±SD	41.8±8.7
Median (IQR)	42.0 (35.0-47.0)
Min-max	18-73
BMI (kg/m²)	
Mean±SD	28.9±6.4
Median (IQR)	27.7 (24.8-32.0)

Regarding the clinical characteristics of lipedema, the distribution across disease stages revealed that stage 2 was the most prevalent (32.2%; n=205), followed by stage 1 (17.7%; n=113) and stage 3 (16.6%; n=106), with stage 4 representing 3.3% (n=21); 12.4% (n=79) reported "Do not know". The most common lipedema type was type III, affecting the hips, thighs, and calves (58.1%; n=285), followed by type II involving the hips and thighs (32.9%; n=161). A substantial proportion of participants reported comorbidities, with hypothyroidism being the most frequent (28.1%; n=179), followed by anxiety/depression (22.6%; n=144) and hypertension (18.5%; n=118). The burden of associated conditions was considerable, with a median of two comorbidities per participant. The clinical characteristics of lipedema in our sample are detailed in Table 2.

**Table 2:** Clinical characteristics and disease severity of lipedema (n=637) Data are presented as n (%) for categorical variables and mean±SD for continuous variables. Percentages may not total 100% due to missing data or rounding. SD: standard deviation

Characteristic	n (%)
Confirmed diagnosis	
Yes	491 (77.1%)
Suspected	131 (20.6%)
No	15 (2.4%)
Lipedema stage	
Stage 2	205 (32.2%)
Stage 1	113 (17.7%)
Stage 3	106 (16.6%)
Do not know	79 (12.4%)
Stage 4	21 (3.3%)
Symptom score (0-8)	
Mean±SD	6.07±1.54

The pattern of hormonal contraceptive use in the study population demonstrated that 588 participants (92.3%) had current or previous experience with hormonal contraception. Among those with contraceptive experience, the most frequently reported contraceptive type was oral pill (59.7%; n=380), followed by hormonal IUD (13.7%; n=87), oral pill + hormonal IUD (5.5%; n=35), injection (2.7%; n=17), and implant (2.2%; n=14); participants could report more than one contraceptive type over time.** **The median duration of use was 7 years (IQR: 3-15 years), with a mean of 10.3±9.1 years, indicating substantial long-term exposure to exogenous hormones in this population. The age at onset of contraceptive use averaged 20.7±5.6 years, typically coinciding with the period of reproductive maturity. Among current users, continuous use patterns predominated (67.4%), while 32.6% reported interrupted use with periods of discontinuation. The comprehensive pattern of hormonal contraceptive use is presented in Table 3.

**Table 3:** Pattern and type of hormonal contraceptive use among the study participants (n=637) *Duration of use reported for 588 participants with current or previous contraceptive experience. †Only the five most frequently reported contraceptive types are shown; multiple contraceptive types could be reported by participants who used different methods over time. IUD: intrauterine device

Variable	n (%)
Use status	
Previous user	382 (60%)
Current user	230 (36.1%)
Never used	25 (3.9%)
Contraceptive type*†	
Oral pill	380 (59.7%)
Hormonal IUD	87 (13.7%)
Oral pill + hormonal IUD	35 (5.5%)
Injection	17 (2.7%)
Implant	14 (2.2%)

**Figure 1:**
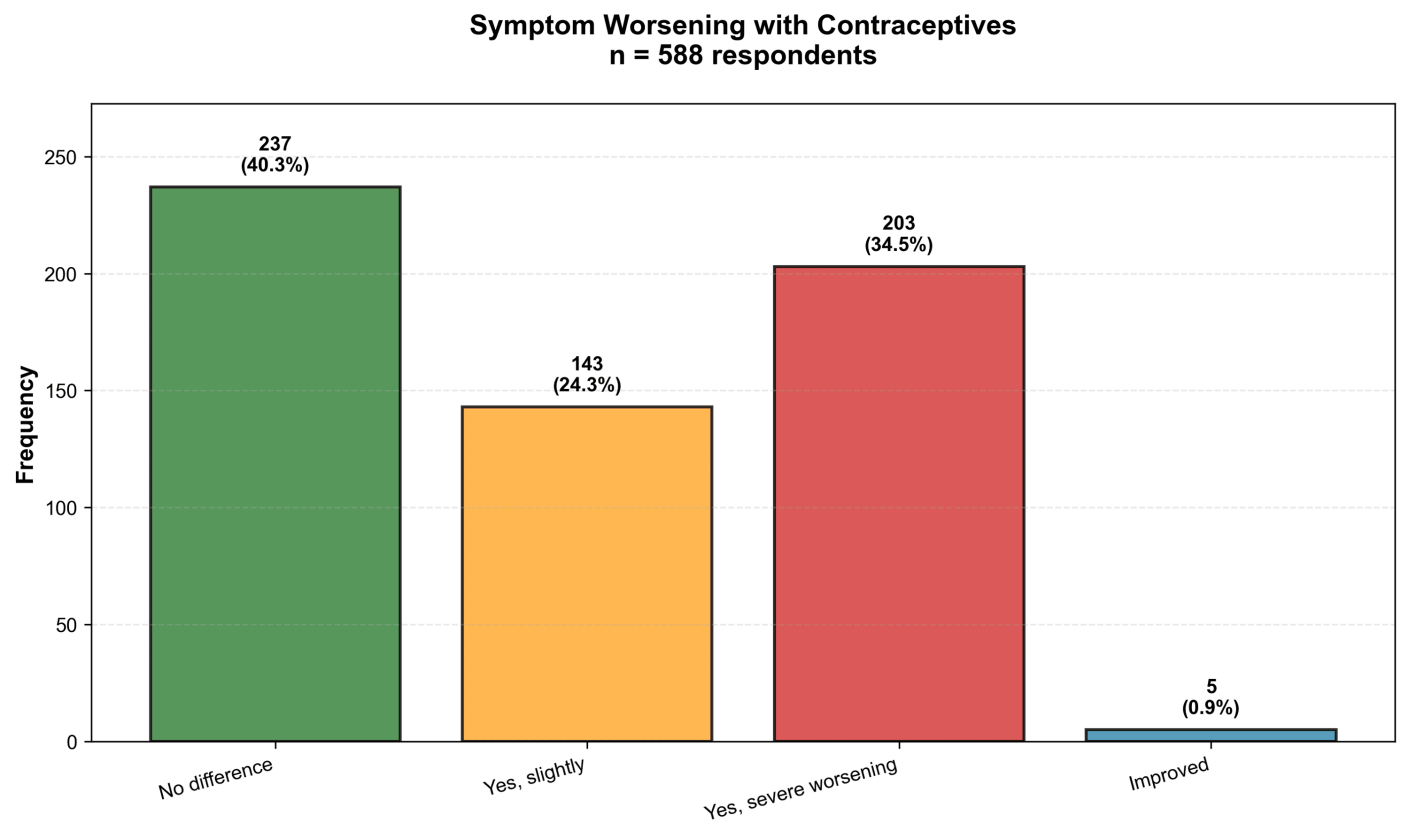
Distribution of perceived lipedema symptom changes with hormonal contraceptive use (n=588) The majority of participants (58.8%) reported some degree of worsening: 34.5% severe and 24.3% slight. Only 40.3% noticed no difference, and 0.9% reported improvement.

One of the most clinically significant findings of this study was the substantial prevalence of lipedema symptom worsening associated with hormonal contraceptive use. Among the 588 participants who had used contraceptives, 346 (58.8%) reported experiencing some degree of symptom exacerbation. Specifically, 203 participants (34.5%) reported severe worsening, while 143 (24.3%) noted slight worsening. Only 237 participants (40.3%) perceived no difference in symptoms, and remarkably, only five participants (0.9%) reported improvement. This distribution of responses is detailed in Table 4 and visually represented in Figure 1. The statistical significance of this association was confirmed through chi-squared testing, which demonstrated that the distribution of responses differed markedly from the expected uniform distribution (χ²=213.71; p<0.001), definitively indicating that the perception of worsening was not a random phenomenon.

**Table 4:** Distribution of perceived lipedema symptom changes associated with hormonal contraceptive use (n=588) Data represent responses from 588 participants with current or previous contraceptive experience. Total reporting any degree of worsening: 346 (58.8%). The distribution differs significantly from uniform expectation (χ²=213.71; p<0.001).

Response	n (%)
No difference noticed	237 (40.3%)
Yes, severe worsening	203 (34.5%)
Yes, slightly	143 (24.3%)
Improved	5 (0.9%)

**Figure 2:**
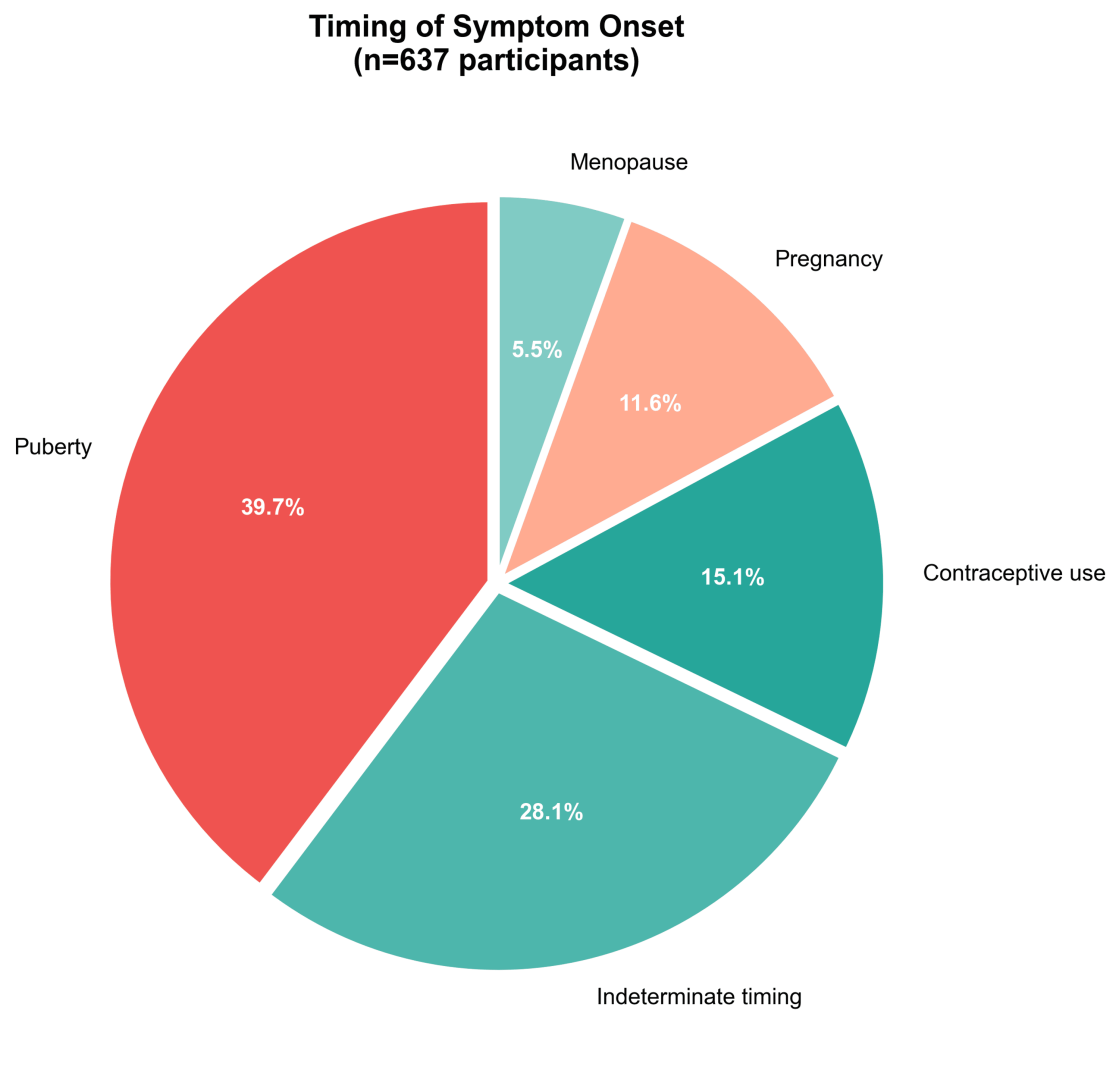
Temporal distribution of lipedema symptom onset (n=637) Puberty was the most common period of initial manifestation (39.7%), followed by indeterminate timing (28.1%). Contraceptive use (15.1%), pregnancy (11.6%), and menopause (5.5%) were also reported as trigger periods.

A particularly intriguing temporal finding emerged from the free-text analysis using natural language processing. When participants were asked about the timing of lipedema symptom onset, 15.1% (n=96) specifically reported that their symptoms began concurrently with the initiation of contraceptive use, suggesting a possible temporal association that warrants further prospective investigation. This finding is depicted in Figure 2, which shows that while puberty remained the most common period of symptom onset (39.7%; n=253), contraceptive use represented the third most frequently reported trigger period, following indeterminate timing (28.1%; n=179) and preceding pregnancy (11.6%; n=74) and menopause (5.5%; n=35).

**Figure 3:**
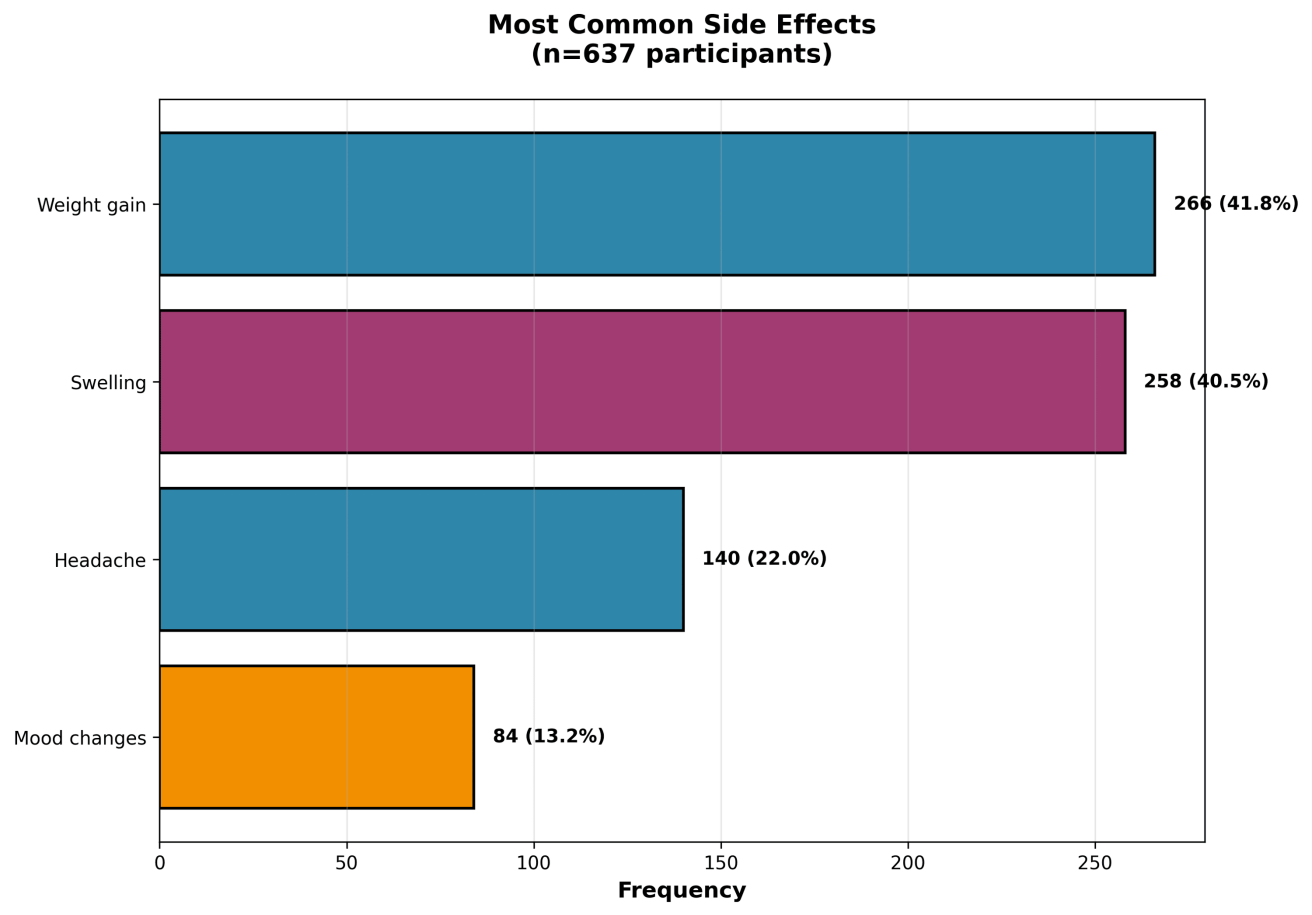
Prevalence of the most commonly reported side effects during hormonal contraceptive use (n=637) Weight gain (41.8%) and swelling (40.5%) were the most prevalent adverse effects, followed by headache (22%) and mood changes (13.2%).

To better understand the mechanisms underlying contraceptive-associated worsening, we analyzed the relationship between specific side effects and symptom exacerbation. The most commonly reported side effects were weight gain (41.8%; n=266), swelling (40.5%; n=258), headache (22%; n=140), and mood changes (13.2%; n=84), as illustrated in Figure 3. Importantly, these side effects showed significant associations with lipedema worsening. Women who experienced weight gain as a contraceptive side effect were substantially more likely to report lipedema worsening (71.7%) compared to those without weight gain (43.5%), representing a clinically meaningful difference of 28.2 percentage points (χ²=29.32; p<0.0001). Similarly, mood alterations as a side effect were associated with higher rates of symptom worsening (77.4% versus 51.3% in those without mood changes; χ²=14.23; p=0.0002).

The impact of lipedema on quality of life was substantial and objectively quantifiable. The pain scale (ranging from 0 to 10) showed a mean score of 5.2±2.6, with a median of 5.00, indicating moderate to severe chronic pain in this population. The quality of life impact score (ranging from 0 to 15) demonstrated a mean of 3.09±1.89, with a median of 3.00. Self-esteem assessment revealed considerable psychosocial burden, with 70.5% of participants reporting either fair (38.3%; n=244) or low (32.2%; n=205) self-esteem, while only 22.3% reported good or excellent self-esteem. These findings are summarized in Table 5.

**Table 5:** Pain intensity, quality of life impact, and self-esteem among the study participants Pain scale ranges from 0 (no pain) to 10 (maximum pain) [10,11]. Quality of life impact score ranges from 0 (no impact) to 15 (maximum impact), calculated as weighted sum of mobility limitations, work impact, social impact, emotional impact, and daily activity difficulties [10]. Sample sizes vary due to incomplete responses: pain scale n=559, quality of life impact score n=310, and self-esteem n=591. SD: standard deviation

Variable	Value
Pain scale (0-10)	
Mean±SD	5.2±2.6
Median	5.00
Quality of life impact score (0-15)	
Mean±SD	3.09±1.89
Median	3.00
Self-esteem, n (%)	
Fair	244 (38.3%)
Low	205 (32.2%)
Good	128 (20.1%)
Excellent	14 (2.2%)

Correlation analyses revealed several statistically significant relationships among key variables (Table 6). As expected, age showed a very strong correlation with years since menarche (r=0.973; p<0.0001). More clinically relevant, BMI demonstrated a moderate positive correlation with quality of life impact score (r=0.313; p<0.0001), suggesting that higher BMI is associated with greater functional impairment. The symptom score showed a moderate correlation with the pain scale (r=0.431; p<0.0001), and notably, the pain scale itself exhibited the strongest correlation with quality of life impact (r=0.500; p<0.0001), indicating that pain intensity is a primary driver of functional limitations in women with lipedema.

**Table 6:** Significant correlations among key clinical variables (Spearman rank correlation) Only correlations with r>0.3 and p<0.05 are shown. Correlation strength interpretation: r=0.3-0.5 (moderate); r=0.5-0.7 (strong); and r>0.7 (very strong). BMI: body mass index; F6: question F6 from study questionnaire (pain scale 0-10)

Variable 1	Variable 2	r	P-value	n
Age	Years since menarche	0.973	<0.0001	637
BMI	Quality of life impact score	0.313	<0.0001	637
Symptom score	Pain scale (F6)	0.431	<0.0001	559
Pain scale (F6)	Quality of life impact score	0.500	<0.0001	559

To identify independent predictors of lipedema worsening associated with contraceptive use while controlling for potential confounders, we conducted multivariate logistic regression analysis with 568 participants who had complete data. The model demonstrated modest overall fit (Pseudo R²=0.048; Akaike information criterion (AIC)=752.0; Bayesian information criterion (BIC)=782.4), suggesting that contraceptive-associated worsening is influenced by multiple factors beyond those measured in this study. The results, presented in Table 7, revealed that the symptom score was the strongest independent predictor of worsening (OR=1.562; 95% CI: 1.300-1.877; p<0.0001), indicating that each one-point increase in baseline symptom severity was associated with a 56% increase in the odds of experiencing contraceptive-related worsening. This finding suggests that women with more severe pre-existing symptoms are particularly vulnerable to hormonal exacerbation.

**Table 7:** Multivariate logistic regression model for predictors of lipedema worsening with contraceptive use (n=568) Dependent variable: any degree of symptom worsening (yes/no) during contraceptive use. All continuous variables were standardized prior to analysis. Model fit: Pseudo R²=0.048; AIC=752.0; BIC=782.4. *p<0.05. Sample includes only participants with complete data on all model variables. OR: odds ratio; CI: confidence interval; BMI: body mass index; AIC: Akaike information criterion; BIC: Bayesian information criterion

Variable	OR	95% CI	P-value
Age	0.926	0.776-1.105	0.3949
BMI	0.819*	0.680-0.988	0.0369
Age at menarche	0.746*	0.591-0.941	0.0135
Duration of use (years)	0.931	0.789-1.100	0.4018
Symptom score	1.562*	1.300-1.877	<0.0001
Number of comorbidities	0.904	0.759-1.076	0.2572

Interestingly, two other variables showed significant but protective associations. Age at menarche demonstrated a protective effect (OR=0.746; 95% CI: 0.591-0.941; p=0.0135), suggesting that women with later onset of menstruation may have reduced susceptibility to contraceptive-associated worsening. BMI also showed a modest protective association (OR=0.819; 95% CI: 0.680-0.988; p=0.0369), a somewhat counterintuitive finding that may reflect selection bias or unmeasured confounding and warrants further investigation. Notably, the duration of contraceptive use was not significantly associated with worsening (OR=0.931; 95% CI: 0.789-1.100; p=0.4018), suggesting that symptom exacerbation may be related to individual susceptibility rather than cumulative hormonal exposure. Neither age nor number of comorbidities showed significant associations with worsening in the adjusted model.

To complement our understanding of the functional impact of lipedema, we performed multiple linear regression to identify predictors of quality of life impairment in 310 participants with complete data. The model explained 29.5% of the variance in quality of life scores (R²=0.295; adjusted R²=0.281; F(6,303)=21.16; p<0.001). As shown in Table 8, the pain scale emerged as the dominant predictor (β=0.641; 95% CI: 0.454-0.827; p<0.0001), with standardized coefficients indicating that pain intensity was by far the most influential factor affecting quality of life. BMI was the second significant predictor (β=0.364; 95% CI: 0.194-0.535; p<0.0001), suggesting that body composition independently contributes to functional limitations beyond its correlation with symptom severity. Notably, the degree of worsening with contraceptives did not emerge as a significant predictor of quality of life impact after adjustment for confounding factors (β=0.106; 95% CI: -0.054 to 0.266; p=0.1943), suggesting that hormonal effects on symptoms may influence quality of life through complementary pathways mediated primarily by pain and body composition rather than through direct independent effects.

**Table 8:** Multiple linear regression model for predictors of quality of life impact (n=310) Dependent variable: quality of life impact score (0-15 points). All continuous variables were standardized prior to analysis. Model fit: R²=0.295; adjusted R²=0.281; F(6,303)=21.16; p<0.001. *p<0.05. Sample includes only participants with complete data on all model variables. β: standardized regression coefficient; CI: confidence interval; BMI: body mass index

Variable	β (standardized)	95% CI	P-value
Age	0.061	-0.105 to 0.226	0.4718
BMI	0.364*	0.194 to 0.535	<0.0001
Symptom score	0.044	-0.137 to 0.225	0.6346
Pain scale (F6)	0.641*	0.454 to 0.827	<0.0001
Degree of worsening with contraceptive	0.106	-0.054 to 0.266	0.1943
Number of comorbidities	0.078	-0.085 to 0.240	0.348

In summary, these findings demonstrate a significant and clinically relevant association between hormonal contraceptive use and lipedema symptom worsening in a substantial majority of users, with symptom severity, age at menarche, and specific side effects (particularly weight gain and mood changes) serving as important modulating factors. The impact on quality of life is primarily mediated through pain intensity and BMI, with chronic pain representing the most important modifiable target for therapeutic intervention. The temporal coincidence of symptom onset with contraceptive initiation in 15.1% of participants, combined with the high prevalence of perceived worsening, suggests that exogenous hormones may play an important role in both the triggering and progression of lipedema manifestations, although the cross-sectional design precludes definitive causal inference.

## Discussion

This study, based on the largest Brazilian sample investigated on lipedema (n=637), provides robust and clinically relevant evidence of a significant association between hormonal contraceptive use and the worsening of lipedema symptoms. This high prevalence of perceived worsening, statistically significant (p<0.001), corroborates previous reports in the literature [2,4,5] and transforms a frequent clinical observation into a measurable scientific finding, suggesting that exogenous hormones play an important role in the pathogenesis and progression of lipedema [1].

It is fundamental, in the analysis of these results, to differentiate direct statistical associations from broader theoretical interpretations. The data from the present study firmly support the main association: a high prevalence (58.8%) of the perception of lipedema symptom worsening associated with hormonal contraceptive use. Similarly, regressions robustly identify the pain scale (β=0.641) and BMI (β=0.364) as the dominant predictors of negative impact on quality of life. However, broader explanatory theories, particularly the "inflammatory model", should be treated as speculative [1,12-14]. The interpretation that the absence of correlation with duration of use (p=0.4018) suggests an "individual susceptibility" instead of a cumulative effect is a working hypothesis, not a proven conclusion, since the study did not measure objective inflammatory or lymphatic biomarkers. This clear distinction between statistical association and causal hypothesis is crucial for the correct interpretation of the findings.

These findings reinforce the hypothesis that lipedema is not only modulatable by hormonal factors but also deeply influenced by inflammatory and lymphatic pathways, in which contraceptives act as contextual amplifiers.

An interesting finding was that 15.1% of participants (n=96) reported that the onset of lipedema symptoms coincided temporally with the start of contraceptive use. Although this finding suggests a possible temporal association, one of the Bradford Hill [15] criteria for causality, it is important to emphasize that in cross-sectional studies it is not possible to distinguish between (a) contraceptives as a triggering factor, (b) unmasking of pre-existing subclinical disease, and (c) recall bias. Prospective studies are necessary to clarify this relationship. When combined with the strong dose-response association observed between symptom score and risk of worsening (OR=1.562; 95% CI: 1.300-1.877; p<0.0001), where each additional symptom increased the risk of worsening by 50%, the hypothesis of a relationship possibly mediated by plausible biological mechanisms is strengthened [16,17], even without direct causal confirmation.

A relevant finding was that the duration of contraceptive use did not predict worsening (OR=0.931; p=0.4018), nor did age (OR=0.926; p=0.3949), indicating that the adverse response does not follow a cumulative dose-dependent pattern. Significant predictors were baseline symptom score (OR=1.562; p<0.0001), suggesting that pre-existing severity determines vulnerability to hormones, while age at menarche (OR=0.746; p=0.0135) and BMI (OR=0.819; p=0.0369) showed modest protective effects, possibly reflecting differences in metabolic-hormonal programming. A possible interpretation is that worsening with contraceptives may reflect individual susceptibility rather than a dose-dependent effect, where predisposed women manifest worsening early while non-susceptible ones tolerate prolonged use. Alternatively, this finding may reflect survival bias (women who worsened significantly discontinued use early) or insufficient statistical power to detect weak associations. The fact that baseline symptom score was the strongest predictor (OR=1.562; p<0.0001) suggests that the pre-existing severity of the condition may influence the response to exogenous hormones, although the exact mechanism remains to be elucidated. This explains why the strongest predictor was precisely the baseline symptom score (OR=1.562; p<0.0001), a direct marker of pre-existing inflammatory activity, and not the duration of exposure. Age at menarche (OR=0.746; p=0.0135), in turn, acts as a temporal proxy for immunometabolic programming: early menarche implies a larger window of inflammatory "imprinting" during tissue maturation, establishing elevated baseline reactivity. In summary, lipedema associated with contraceptives seems to emerge from constitutive vulnerability amplified by hormones, not toxic accumulation, a paradigm that reorients clinical focus from duration of use to the early identification of risk phenotypes.

A counterintuitive finding deserves highlighting: the duration of contraceptive use was not a significant predictor of worsening in the multivariate analysis (OR=0.931; p=0.4018). If worsening were a cumulative dose-dependent phenomenon, we would expect longer duration of use to correlate with a higher probability of deterioration. The absence of this relationship suggests an alternative mechanism: instead of a cumulative effect, there seems to be individual susceptibility, where vulnerable women manifest worsening early (possibly in the first few cycles), while non-susceptible ones may use contraceptives indefinitely without significant deterioration.

The analysis of side effects revealed a strong association between weight gain and lipedema worsening (χ²=29.32; p<0.0001): 71.7% of women who experienced weight gain reported symptom worsening, compared to 43.5% of those without weight gain, a difference of 28.2 percentage points. This finding admits multiple interpretations. First, metabolic and fluid retention effects of contraceptives may exacerbate pre-existing lipedema symptoms, creating an overlap between iatrogenic side effects (hormone-induced edema) and manifestations of the underlying disease [18-21]. Second, weight gain may reflect a marker of individual hormonal response, where women more metabolically sensitive to contraceptives also present greater reactivity in the adipose tissue affected by lipedema [22-24]. Third, reverse causality cannot be excluded, where lipedema worsening (with increased edema and tissue volume) is interpreted by participants as "weight gain". The cross-sectional design of the study does not allow distinguishing between these hypotheses, although all are biologically plausible and not mutually exclusive.

Mood alteration as a side effect also showed a significant association with lipedema worsening (77.4% versus 51.3%; p=0.0002). This finding can be interpreted in different ways. One possibility is that mood changes reflect increased sensitivity to the systemic effects of exogenous hormones, potentially indicating greater individual susceptibility both in the central nervous system and in other target tissues, including adipose tissue [25,26]. Alternatively, women with more severe lipedema symptoms may present a higher baseline psychological stress load, making them more vulnerable to contraceptive-induced mood changes, representing confounding rather than direct causality [3]. Finally, both mood symptoms and lipedema worsening may be influenced by unmeasured variables (such as stress, sleep, and dietary factors), creating a spurious association. Prospective studies with standardized psychometric assessment and biological markers of hormonal sensitivity are necessary to elucidate the nature of this association.

The magnitude of the observed association is clinically significant: among 588 hormonal contraceptive users, 58.8% reported symptom worsening (χ²=213.71; p<0.001), a distribution that differs significantly from random expectation. Particularly relevant, 15.1% of participants (n=96) reported temporal coincidence between the start of contraceptives and symptom onset, suggesting a possible temporal association that merits prospective investigation.

Although the present study did not include objective measures of inflammation or lymphatic assessment, the findings are remarkably consistent with emerging pathophysiological models in the literature that propose lipedema as a chronic inflammatory syndrome of soft tissues. Several authors have suggested an integrated inflammation → lymphatic → adipose model, in which low-grade inflammatory processes precede and perpetuate the characteristic changes of the disease [27,28].

In this conceptual framework, mast cell activation, vascular permeability alterations, progressive lymphatic dysfunction, and the release of inflammatory mediators (histamine, tryptase, prostaglandins, cytokines) would create a microenvironment conducive to disproportionate adipocyte hyperplasia and fibrosis [28-33]. Recent multi-omics studies have identified characteristic molecular signatures, including polarized M2 macrophages, elevated CD163 expression, interstitial hypoxia, and extracellular matrix remodeling mediated by metalloproteinases [13,14,34].

These findings refine our understanding of the "Immunological Shield", suggesting that the protection conferred by lipedema tissue comes at a homeostatic cost. We know from the "Menopausal Switch" concept that estrogen is vital for maintaining the "Metabolic Sink's" protective function against cancer. However, the introduction of synthetic hormones appears to create a paradox: they maintain the "systemic protection" signal but overwhelm the local vascular and lymphatic drainage capacity. Therefore, the hormone is not the villain, but a potent modulator that, in a predisposed phenotype, can tip the balance between systemic immunometabolic advantage and local symptomatic comfort, necessitating a personalized clinical approach rather than the demonization of female endocrinology [14,34-36].

Connection between the study findings and the inflammatory model

Several findings from the present study, while not proving this model, are consistent with its predictions:

First, the absence of an effect of duration of use (OR=0.931; p=0.4018) contradicts the cumulative hormonal toxicity model [37-39] and suggests individual susceptibility, consistent with the hypothesis that women with already elevated tissue "inflammatory tone" respond adversely from the beginning of exposure, regardless of duration.

Second, the baseline symptom score as the dominant predictor of worsening (OR=1.562; p<0.0001) suggests that pre-existing disease activity, a possible indirect marker of underlying inflammatory process, determines vulnerability to exogenous hormones.

Third, the protective association of age at menarche (OR=0.746; p=0.0135) could reflect differences in inflammatory "programming" during critical developmental windows, although alternative interpretations (confounding, chance) cannot be excluded [40-43]. The hypothesis would be that later menarche implies less cumulative exposure to estrogenic fluctuations during the maturation of immune and vascular systems in subcutaneous adipose tissues.

Fourth, the most prevalent side effects, namely, swelling (40.5%) and weight gain (41.8%), are expected manifestations of both inflammatory fluid retention and direct hormonal effects, making the distinction between cause and consequence difficult [18-21,44,45].

Hormones as modulators, not causers

This framework suggests that sex hormones, whether endogenous or exogenous, would not be the primary cause of lipedema, but gain modulators that amplify pre-existing inflammatory vulnerability. This would explain why milestones of intense hormonal variation (puberty, pregnancy, contraceptives, menopause) frequently coincide with symptom onset or exacerbation, without being direct causers [3,46]. Estrogens and progestogens would modulate mast cells, endothelium, and lymphatic function, amplifying circuits already predisposed to dysfunction [47-49].

From an evolutionary medicine perspective, it is speculated that lipedema may represent a maladaptive response of ancestral protective peripheral reserve programs when confronted with modern environments characterized by chronic inflammatory exposure (pro-inflammatory diet, sedentary lifestyle, environmental endocrine disruptors, sleep deprivation). In this context, continuous exogenous hormonal exposure would act as an amplifier of an already inflammosensitive phenotype [50].

Critical limitations and need for validation

It is imperative to emphasize that these interpretations are speculative and based on literature synthesis, not direct measures. The present study did not measure inflammatory biomarkers (CRP, IL-6, TNF-α, tryptase), did not objectively assess lymphatic function (lymphoscintigraphy, indocyanine green (ICG)), and did not perform histopathological or molecular analysis. Furthermore, this study cannot distinguish between genuine pathophysiological worsening of lipedema and independent side effects (iatrogenic edema) interpreted as disease worsening.

Therefore, the inflammatory model remains an attractive and biologically plausible hypothesis, but not validated by the data of the present study.

The findings not only elucidate etiological aspects but also have direct implications for clinical practice and the design of therapeutic interventions. Before starting contraceptives, clinicians should actively investigate signs of lipedema using validated screening tools integrated into routine gynecological protocols. Women with confirmed or suspected lipedema should receive explicit information about the risk of worsening to allow for an informed choice between hormonal contraceptives and non-hormonal methods. In women who experience worsening, evidence-based adjuvant measures for chronic inflammatory conditions may be considered, including nutritional optimization, exercises with lymphatic emphasis, and lifestyle factor management. Furthermore, longitudinal research testing inflammatory reversibility and pragmatic trials comparing strategies centered on inflammatory control are necessary. Finally, early and objective monitoring is recommended for women who choose to continue contraceptives despite the risk.

Our questionnaire did not capture progestogen type in sufficient detail to support subgroup analyses by specific formulations; therefore, we cannot draw any conclusions about individual progestogens from our data. Nevertheless, when choosing a contraceptive for women with chronic edema and pain, such as those with lipedema, clinicians should take into account the pharmacological profile and known vascular risks of each formulation. In this context, drospirenone-containing combined oral contraceptives deserve particular caution, given their distinct antimineralocorticoid and antiandrogenic actions and the higher venous thromboembolism risk reported in comparative studies, even though these aspects were not directly evaluated in the present analysis. Robust evidence from large cohort studies and meta-analyses demonstrates that combined oral contraceptives containing drospirenone are associated with a venous thromboembolism risk approximately 1.5-3 times higher compared with second-generation formulations containing levonorgestrel [51-53]. Considering that women with lipedema frequently present elevated baseline risk factors, such as increased BMI, venous stasis, and low-grade chronic inflammation, the addition of a formulation with greater thrombogenic potential may represent a clinically unnecessary increase in risk [3,29,54]. Therefore, in women with lipedema who require hormonal contraception, it seems prudent to avoid drospirenone-containing preparations when safer alternatives are available and to prioritize formulations with a more favorable hemostatic profile, such as levonorgestrel-containing combined pills, or non-hormonal methods.

The impairment of quality of life was evident and quantifiable in the studied sample. Multivariate linear regression analysis identified that the main independent predictors of greater impact on quality of life were the pain scale (β=0.641; p<0.0001) and BMI (β=0.364; p<0.0001). Notably, the degree of worsening with contraceptives did not emerge as a significant predictor after adjustment for confounding factors (β=0.106; p=0.1943), suggesting that the impact on quality of life is primarily mediated by pain intensity and body composition, regardless of the perception of hormonal worsening.

The psychosocial impact was equally striking, with 70.5% of participants reporting low or fair self-esteem and a median pain score of 5 on a 0-10 scale. These data highlight the double burden imposed by lipedema, physical and emotional, which may be exacerbated by hormonal contraceptive use, reinforcing the need for a comprehensive approach in the management of these patients [3,55,56].

An important methodological differentiator of this study was the innovative use of natural language processing via a local large language model to systematically categorize 19 free-text variables, including side effects, treatments, comorbidities, and timing of symptom onset. This approach allowed the quantification of qualitative data that would traditionally be lost in conventional statistical analyses or require intensive manual categorization, representing a significant methodological advance for lipedema research. The precise identification that 15.1% of women associated symptom onset specifically with hormonal contraceptive use was made possible by this technique, highlighting its value in capturing important clinical nuances.

From an evolutionary perspective, lipedema may represent the modern maladaptation of an ancient protective peripheral reserve program that, in environments of inflammatory ultra-exposure (pro-inflammatory diet, dysbiosis and metabolic endotoxemia, environmental endocrine disruptors, sleep deprivation/chronodisruption, sedentary lifestyle), remains chronically activated [50]. Environmental xenoestrogens, such as bisphenol A and phthalates, act as co-factors, mimicking estrogen and exacerbating endothelial and mast cell dysfunction [57-59]. Continuous exogenous hormonal exposure acts as a modulator that exacerbates this inflammatory-lymphatic phenotype, not necessarily as its initiator. In this frame, lipedema emerges as a phenotype of programmed inflammatory vulnerability, upon which hormones operate as contextual amplifiers. Resistance to muscle mass gain and weight loss through exercise, frequently mentioned by patients, reflects that the tissue is not metabolically normal fat, but an inflamed and fibrotic matrix resistant to conventional lipolysis [60,61].

In practical terms, our findings indicate that among women with lipedema who use hormonal contraceptives, those with a higher baseline symptom burden are more likely to report subsequent worsening, even after adjustment for age, BMI, and comorbidities. Although the cross-sectional design precludes causal inference, this consistent association suggests that a more inflammatory or pain-sensitized phenotype may be particularly vulnerable to exogenous hormonal modulation. The immediate clinical implication is that contraceptive counseling in lipedema should be individualized: women with very high baseline symptom scores should be explicitly informed about the possibility of worsening, monitored more closely after initiation, and offered non-hormonal alternatives when appropriate. Looking ahead, if future prospective studies incorporating objective inflammatory markers confirm that specific inflammatory endotypes predict symptom aggravation with contraceptives, targeted pre-prescription assessment, combining clinical symptom scores with selected biomarkers such as hs CRP or baseline tryptase, could help identify women at higher risk and guide a more personalized choice of contraceptive methods. Multimodal anti-inflammatory interventions could also be tested in future trials, including strategies combining anti-inflammatory nutrition, mast cell stabilizers such as ketotifen or cromolyn, targeted supplementation including curcumin, quercetin, or palmitoylethanolamide, sleep optimization, and lymphatic-focused exercise [62-68]. Objective monitoring through biomarker tracking before and after interventions would be essential to assess reversibility, and risk stratification could be further enhanced by developing predictive scores that integrate baseline symptoms, hormonal history, and inflammatory markers.

This study presents limitations that must be considered. First, the cross-sectional design does not allow for establishing definitive causality, only temporal association. Second, data are self-reported, without objective validation by anthropometric measures, inflammatory biomarkers, or imaging exams, making it impossible to categorically distinguish between genuine pathophysiological worsening of lipedema and independent side effects (iatrogenic fluid retention) interpreted as worsening of the underlying disease. Third, recall bias may affect memory regarding the timing of symptom onset and medication use. Fourth, a convenience sample recruited from support groups may overestimate the association (selection bias). Finally, it was not possible to stratify by specific type of contraceptive, dosage, or route of administration. Despite these limitations, the large sample size (n=637), robust multivariate analyses, and magnitude of the observed effect (58.8%) confer substantial validity to the findings.

It is worth acknowledging an important methodological limitation: without objective measures (inflammatory biomarkers, lymphoscintigraphy, soft tissue ultrasonography), it is not possible to categorically distinguish between (A) genuine pathophysiological worsening of lipedema, (B) independent side effects of contraceptives (iatrogenic fluid retention) erroneously interpreted as worsening of the underlying disease, and (C) hypervigilance bias in women with already established severe symptoms. Future studies should incorporate objective endpoints, that is, variation in standardized circumferences, body water index by bioimpedance, serum tryptase dosage, and vascular permeability markers, to separate real inflammatory signal from perceptual noise. This caveat, however, does not invalidate the observed association pattern, only nuances its mechanistic interpretation. Still, the consistency of findings across multiple analyses, the temporal coherence, and the magnitude of observed effects confer epidemiological credibility to the identified pattern.

Prospective studies should follow women before the start of contraceptives with objective measures (circumferences, bioimpedance, inflammatory biomarkers) to establish temporal causality. Controlled interruption trials are needed to test symptom reversibility after hormonal discontinuation, ideally also comparing multimodal anti-inflammatory interventions in a factorial design. Molecular characterization via subcutaneous tissue biopsy and pharmacogenomic studies could identify susceptibility phenotypes and precise therapeutic targets. Finally, stratification by type, dose, and route of administration of contraceptives would allow identifying hormonal profiles with less deleterious impact in women with lipedema.

## Conclusions

This study demonstrated a significant and clinically relevant association between hormonal contraceptive use and the worsening of lipedema symptoms. The findings are consistent with the hypothesis that exogenous hormones may modulate the clinical expression of the disease in predisposed women. Although the exact mechanisms remain to be elucidated, the data suggest that baseline symptom score and reproductive history may influence individual response to contraceptives. These findings have immediate implications for contraceptive counseling of women with lipedema, while prospective studies with objective measures are needed to establish causality and mechanisms.

## Appendices

**Table 9:** Study questionnaire IUD: intrauterine device; BR: Brazil

Section	Question	Type/options
A. Consent and ID	A1. Consent	Checkbox (required)
	A2. Age	Number (18-100)
	A2.1 Current weight (kg)	Decimal number
	A2.2 Height (cm)	Number
	A2.3 State of residence	Dropdown (BR states)
B. Screening	B1. Do you feel pain in your legs?	Yes/No/Sometimes
	B2. Do your legs swell during the day?	Yes/No/Sometimes
	B3. Do symptoms worsen with heat?	Yes/No
	B4. Are your legs sensitive to touch?	Yes/No
	B5. Do you bruise easily on your legs?	Yes/No
	B6. Do you notice disproportionate fat accumulation?	Yes/No
	B7. Do diets and exercise fail to reduce leg size?	Yes/No
	B8. Is there a family history of lipedema?	Yes/No/Don't know
	B9. When did you first notice these symptoms?	Puberty/Pregnancy/Menopause/Contraceptive use/Other
C. Diagnosis	C1. Have you been diagnosed with lipedema?	Yes/No/Suspected
	C2. Diagnosis date	Month/Year
	C4. Lipedema stage	Stages 1-4/Don't know
	C5. Lipedema type	Types I-V/Don't know
	C6. Other medical conditions	Long text
D. Hormonal	D1. Age at menarche	Number
	D2. Menstrual cycle regularity	Yes/No/Menopause
	D4. Number of pregnancies	Number
	D6. Menopause status	Yes/No/Perimenopause
	D9. Thyroid alterations	Hypo/Hyper/No
E. Contraceptives	E1. Do you use/have used hormonal contraceptives?	Current/Previous/Never
	E2. Type of contraceptive	Pill/Injection/IUD/Implant/Patch/Ring
	E3. Brand name	Text
	E4. Duration of use	Text
	E5. Age at start of use	Number
	E6. Pattern of use	Continuous/Interrupted
	E9. Side effects noticed	Weight gain/Swelling/Headache/Mood/etc.
	E10. Worsening of lipedema after starting?	Severe/A little/None/Improved
	E12. Weight gain after starting?	Yes/No
F. Quality of life	F1. Impact on mobility	Very much/Moderately/Little/None
	F3. Impact on work	Yes/No/Don't work
	F6. Average daily pain (0-10)	Number (0-10)
	F8. Self-esteem	Excellent/Good/Fair/Low
